# Bioactivity of Synthesized Trifluoromethyl Thioxanthone Analogues

**DOI:** 10.3390/ph18040561

**Published:** 2025-04-11

**Authors:** Murad Abualhasan, Hussein Haider, Ahmad Odeh, Amer Daraghmeh

**Affiliations:** Faculty of Pharmacy, An-Najah National University, Nablus 00970, Palestine; hussienhaidr0597184299@gmail.com (H.H.); ph.ahmad.o.r@gmail.com (A.O.); amersalehdd@gmail.com (A.D.)

**Keywords:** trifluoromethyl thioxanthene, cytotoxicity, anti-amylase, antilipase, antioxidant

## Abstract

**Background:** The study aims to evaluate the potential of trifluoromethyl thioxanthene derivatives across various biological activities, including antioxidant properties, anti-amylase effects, pancreatic lipase inhibition, anticancer activity, and COX inhibition. This research offers insights into the therapeutic applications of these compounds for managing metabolic disorders and inflammation. **Method:** Tertiary alcohols were synthesized using Grignard reagents and subsequently combined with L-cysteine, with their structures confirmed via NMR and IR spectroscopy. **Results:** The results indicated compound **3** exhibited the highest antioxidant potential, with 46.6% at 80 µg/mL in the DPPH assay. Compound **4** showed moderate pancreatic lipase inhibition, exhibiting an IC_50_ range of 100.6 to 277 µM. Compound **1** revealed potent anticancer activity against HeLa cells, with an IC_50_ of 87.8 nM. Compound **2** showed a potent antioxidant and anti-amylase activity with IC_50_ of 1.67 ± 0.5 and 60.2 ± 0.8 µM, respectively. Furthermore, the synthesized compounds **1**, **3**, and **4** displayed promising COX-2 inhibition with IC_50_ values ranging from 6.5 to 27.4 nM, suggesting potential anti-inflammatory benefits. **Conclusions:** This study highlights the significant biological activities of trifluoromethyl thioxanthene derivatives, positioning them as promising candidates for the treatment of cancer, metabolic disorders, and inflammation. These compounds demonstrated noteworthy antioxidant and enzyme inhibition properties, warranting further in vivo studies to confirm their therapeutic efficacy.

## 1. Introduction

Cancer encompasses a group of diseases characterized by the rapid proliferation of abnormal cells that can invade nearby tissues and metastasize to other organs, known as metastasis [1]. Cancer arises from precancerous lesions and is influenced by genetic factors and various carcinogens [2]. With approximately 10 million deaths attributed to cancer annually, early detection is vital for effective treatment, which may involve surgery, chemotherapy, hormonal therapy, or radiation [3,4,5].

Inflammation is the body’s physiological response to foreign organisms, including pathogens and dust [6]. It can be acute or chronic and plays a significant role in various disorders, such as cancer, diabetes, and cardiovascular diseases [7]. Inflammation is a crucial immune response that aids recovery from infections and injuries [8,9,10].

Obesity has emerged as a global health crisis, particularly in the West, significantly affecting health and longevity [11]. It is a complex chronic disease treated with various methods; common anti-obesity drugs include orlistat, which inhibits pancreatic lipase to reduce fat absorption, and sibutramine, which suppresses appetite and increases energy expenditure [12,13].

Certain herbal medicines, such as Citri Reticulate Pericarpium and Mori Radicis Cortex, have shown promise in regulating lipid metabolism [14]. Our research will focus on fat absorption inhibitors and a mammalian alpha-amylase inhibitor to help manage diabetes by reducing glucose absorption post-meal [15]. Diabetes mellitus is characterized by high blood sugar levels due to insufficient insulin production or ineffective insulin response [16,17,18]. Targeting carbohydrate-digesting enzymes, like alpha-amylase and alpha-glucosidase, is crucial for managing this condition [19,20,21].

Synthetic tricyclic compounds, such as imipramine, exhibit inhibitory activity against cancer cells, including prostate cancer; however, the exact mechanisms behind their anticancer effects remain unclear [22]. Studies have demonstrated the anti-inflammatory properties of Abietane derivatives, which serve as precursors to anti-inflammatory agents [23]. The Acenaphthene derivative has also shown antitumor activity [24]. Furthermore, the propellane derivative has been recognized for its anti-proliferative effects against non-small cell lung cancer cell lines [25]. In the case of trifluoromethyl compounds, various studies have reported significant anti-inflammatory activity associated with the incorporation of the trifluoromethyl group [26]. Additionally, these compounds have demonstrated anti-cancer activity against ovarian, lung, breast, and colon cancers [27].

L-cysteine is a semi-essential amino acid [28]. It plays an important role in many processes such as protein folding stability and trafficking, assembly, biosynthesis of coenzyme A, detoxification of heavy metals, and redox balance [29,30]. Inside cells, L-cysteine is the prevailing form due to the highly reducing conditions [31]. The thiol group which present in cysteines makes it a unique amino acid due to the thiol-group in their structure, which can undergo a variety of different nucleophilic reactions and increase its affinity to enzyme-binding sites of metals, such as zinc or iron [32,33]. Thioxanthene, a tricyclic structure, many derivatives of these structures showed anticancer and biological activity [34,35]. Thioxanthone derivatives inhibit topoisomerases, essential enzymes for DNA replication and cell division [36]. The compound TXA1, a thioxanthene derivative, has shown the ability to reduce the viability of breast cancer and melanoma cell lines by modulating autophagy and inducing apoptosis [34].

In a previous study on thioxanthene derivatives, which demonstrated potent antioxidant and cytotoxic effects, we extended our research by introducing an additional trifluoromethyl group to the tricyclic structure [37]. We propose that the trifluoromethyl group will enhance structural rigidity, resulting in reduced conformational flexibility and improved selectivity with fewer side effects. Positioning it at the para site is expected to stabilize a specific conformation while increasing the compound’s lipophilicity. Consequently, this modification may boost the compound’s activity and potentially broaden its impact on other biological systems, such as amylase and protease. To our knowledge, none of the synthesized analogs with a cysteine-coupled thioxanthene core have been evaluated for their anticancer, antioxidant, or anti-inflammatory effects, underscoring the novelty of our work.

This research aims to synthesize a collection of thioxanthene analogs coupled with cysteine and evaluate their biological activity. The anticancer activity of the synthesized analogs was tested against Hela cells. Furthermore, the antioxidant and cyclooxygenase (COX) inhibitory properties of the produced analogs were investigated.

## 2. Results and Discussion

### 2.1. Chemistry

**Scheme 1:**
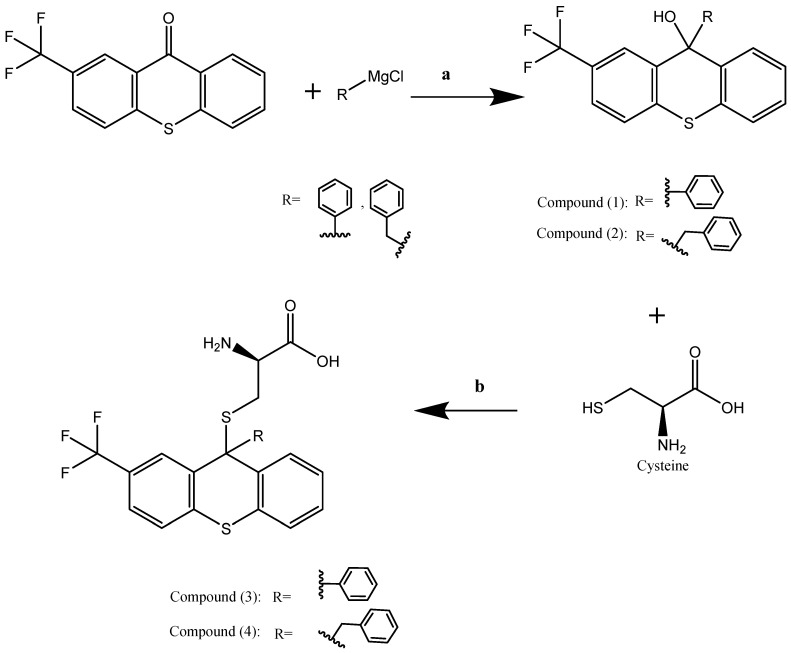
Stepwise Synthesis of compounds **1**–**4**. Reagents and conditions: (**a**) PhMgCl-THF or BzMgCl-THF, DCM, 0 °C to r.t., 24 h (**b**) acetic acid, BF3-Et2O, 0 °C to r.t., 24 h.

All compounds were synthesized according to the methodology outlined in Scheme 1. The trifluoromethyl thioxanthone reagents were employed in the reaction to produce tertiary alcohols, which were subsequently coupled with cysteine. The incorporation of rings was intended to enhance the interaction patterns of hydrophobic and π–π interactions. Additionally, the cysteine moiety was linked to several compounds to actively participate in cancer metabolic remodeling through redox control, thereby augmenting the antioxidant properties of the synthesized structures [38,39]. The synthetic strategy to obtain the final products involved the use of Grignard reagents as alkylating agents for compounds **1** and **2** and BF3 as Lewis acid, which facilitates the coupling of the cysteine moieties to form products 3 and 4.

The products were purified using solvent systems consisting of n-hexane and ethyl acetate. The cysteine-coupled compounds exhibited sharp carbonyl bands around 1600 cm^−1^, while the tertiary alcohols displayed an OH stretch in the vicinity of 3500 cm^−1^. Furthermore, ^1^H-NMR analysis confirmed the chemical structures of the synthesized compounds, revealing a single peak corresponding to one proton for OH in the range of 2.7–2.9 ppm across all tertiary alcohol compounds, alongside multiple signals in the aromatic region. The ^13^C-NMR spectrum indicated a carbonyl signal of around 168 ppm.

### 2.2. Antioxidant Activity Results

The synthesized compounds were evaluated for their antioxidant activity utilizing the DPPH assay. The findings presented in Table 1 revealed varying levels of antioxidant activity among the compounds, with the majority demonstrating moderate efficacy. Interestingly, the compounds exhibited notable potency at lower concentrations, with only slightly increased antioxidant activity at higher concentrations. This behavior may be attributed to the saturation of reactive sites or potential solubility limitations. Compound **3** exhibited an inhibition percentage of 46.6% when tested at a concentration of 80 µg/mL, positioning it as the most potent antioxidant among the compounds synthesized, suggesting significant antioxidant potential. Compounds **4** and **2** also showed good activity, indicating that the cysteine units may contribute to their increased antioxidant activity. In comparison, the positive control, Trolox, demonstrated a superior activity with 98.9% at 80 µg/mL (IC_50_ value of 0.41 ± 0.05 µg/mL), indicating that the antioxidant inhibition of compound **3** is nearly half that of Trolox [40]. Compound **1** exhibits the lowest antioxidant activity, which may be attributed to a cysteine group’s absence and a phenyl group’s presence. The phenyl group is less flexible than the benzyl group found in the more potent antioxidant compound **2**.

In contrast, a study conducted by our team on thioxanthene derivatives revealed that specific compounds exhibited antioxidant activity with more potent antioxidant activity (15.44 to 998.67 nM). This finding indicates that the introduction of a trifluoromethyl group results in lower antioxidant activity, signifying enhanced antioxidant potential compared to the thioxanthene derivatives examined [37].

### 2.3. Results of α-Amylase Inhibition Assay

**Figure 1:**
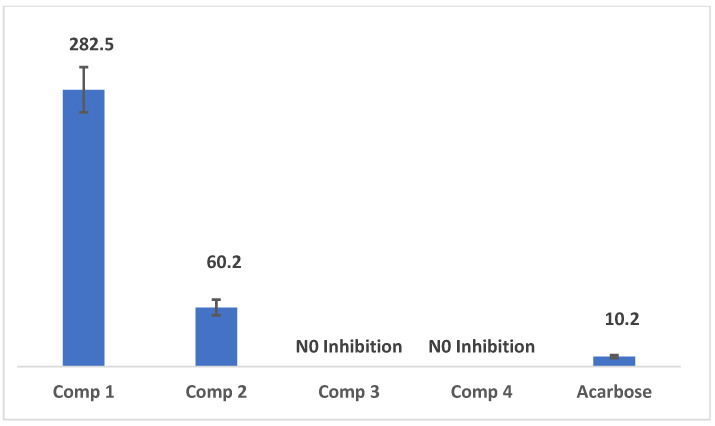
The IC_50_ (µM) of the α-Amylase inhibition activity for the synthesized compounds.

The anti-amylase results indicated moderate activity for compound **2**, a tertiary alcohol with a benzyl group on the trifluoromethyl thioxanthen-9-ol. In contrast, compounds **3** and **4** exhibited no inhibitory effect at all. This suggests that the cysteine substitution on the trifluoromethyl thioxanthen-9-ol reduces its effectiveness. The results were compared to the positive control, acarbose (10.2 ± 0.5 µM), making it approximately six times more potent than compound **2** (60.2 ± 0.8 µM). Detailed results are illustrated in Figure 1. A previous study on an undescribed tricyclic spiroketal compound, purified from the solvent extract of the Microcionidae sponge *Clathria prolifera*, demonstrated inhibitory potential against the carbohydrate-hydrolyzing enzymes α-glucosidase (IC_50_ 0.43 mM) and α-amylase (IC_50_ 0.41 mM) (68). This indicates that our trifluoromethyl thioxanthone, which also possesses a tricyclic structure, displays even more potent anti-amylase activity [41].

### 2.4. Results of Inhibition Assay of Pancreatic Lipase Enzyme

**Figure 2:**
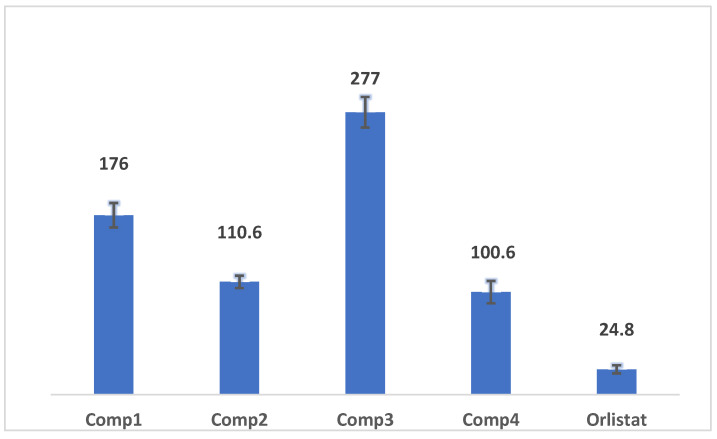
The IC_50_ (µM) of the pancreatic lipase enzyme activity for the synthesized compounds.

The synthesized compounds demonstrated moderate inhibition of lipase activity, with IC_50_ values ranging from 100.6 to 277 µM (Figure 2). Notably, compound **4** exhibited the highest activity, with an IC_50_ of 100.6 ± 7.3 µM, making it four times less potent than the positive control, orlistat. Compound **2** was almost similar to IC_50_ 110.6± 7.5µM. However, compounds **1** and **3** were less active with I50 176.0 and 277.0 µM. A study on C-glycosidic flavones isolated from *Eremochloa ophiuroides* reported IC_50_ values ranging from 18.5 ± 2.6 to 50.5 ± 3.9 µM against pancreatic lipase [42]. In comparison, the synthesized compounds show moderate antilipase activity and are less potent than both Orlistat and specific tricyclic natural products noted in the literature. This suggests structural modifications enhance their inhibitory potency.

### 2.5. Results of the Cytotoxicity Results

**Figure 3:**
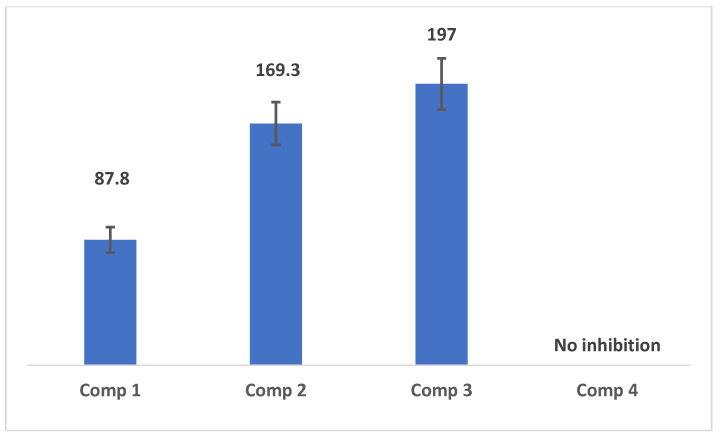
The IC_50_ (nM) of inhibition activity of the synthesized compounds in a concentration range of (100–20,000 nM) against Hela cells.

The anti-cancer activities of the synthesized compounds were tested against HeLa cancer cells. Some of these compounds demonstrated potent cytotoxicity against this specific cancer cell line. Compound **1** exhibited excellent inhibition activity, with an IC_50_ of 87.8 nM against HeLa cells. At a higher concentration of 200 nM, compound **1** showed significant inhibition activity, achieving 93.18% toxicity against HeLa cells. In contrast, compound **4** showed no activity against these cells. Tertiary alcohols, which are compounds lacking cysteine, demonstrated better activity compared to the cysteine-coupled compounds (Compounds **3** and **4**). Notably, compound **1** had the best activity, with an IC_50_ of 87.8 ± 11 nM, which is more potent than the positive control anticancer drug doxorubicin, which has an IC_50_ of 1100 ± 15 nM, when tested against the same cell line [43]. The calculated IC_50_ values for all the synthesized compounds are illustrated in Figure 3. In similar studies involving tricyclic structures, furo [2,3-d]pyrimidinones and pyrrolo [2,3-d]pyrimidinones have exhibited cytotoxic activity against HeLa cells, with IC_50_ values around 6.55 µM [44]. Additionally, crassocolide N, a cembranoid derivative, demonstrated cytotoxicity with an IC_50_ of 4.7 µg/mL [45]. These findings highlight that our synthesized tricyclic compounds, particularly compound **1**, possess superior anticancer potency against HeLa cells compared to other reported structures.

The cytotoxicity tests indicate that the chemicals are effective against the specific cancer cell type being examined. However, it is essential to assess the impact of these active chemicals on other cancer cell lines as well as normal cells, ensuring they exhibit low toxicity. Regrettably, we could not conduct this evaluation due to a shortage of testing supplies during the study. This limitation highlights an important consideration for future research, indicating that such assessments should be included in upcoming studies.

### 2.6. Cyclooxygenase Inhibition Activity

The synthesized drugs were evaluated for their inhibitory activity against the cyclooxygenase isoenzymes COX-1 and COX-2. The results showed that most of the synthesized compounds exhibited good COX-2 inhibition, with IC_50_ values ranging from 6.5 to 27.4 nM. Compound **4** displayed the best IC_50_ for COX-1 inhibition, measuring 10.1 ± 1.3 nM. Furthermore, compounds **1** and **3** demonstrated significant inhibition activity for COX-2, with IC_50_ values of 27.1 ± 0.6 nM and 25.9 ± 0.45 nM, respectively. These results suggest that incorporating a cysteine group into the aromatic tertiary alcohol structure significantly enhances COX-2 inhibition activity. Detailed IC_50_ results can be found in Table 2. Overall, nearly all synthesized compounds were more potent than Celecoxib, a standard positive control, which has an IC_50_ value of 40 nM. However, the positive control showed better selectivity towards COX-2, with a selectivity value of 8.5, compared to the synthesized compounds [46].

## 3. Materials and Methods

### 3.1. Reagents and Materials

All reagents were obtained commercially and used without purification. (2-(Trifluoromethyl)thio-xanthan-9-one) (catalog# AAH6483903, Thermo Fisher Scientific, Massachusetts, MA, USA) was sourced from Fisher. Benzyl magnesium chloride solution (catalog # 302759), phenyl magnesium chloride solution (catalog # 224448), and L-cysteine (catalog #168149) were purchased from Sigma Aldrich, Schnelldorf, Germany. Acetone, methanol, dichloromethane, hexane, and ethyl acetate came from C.S. Company, Haifa, Israel. Diethyl ether (catalog # 38132) was obtained from Merck Millipore, Massachusetts, MA, USA, and tetrahydrofuran (THF) (catalog # 487308) was obtained from Carlo Erba Company, Cornaredo MI, Italy. Sodium sulfate, ammonium chloride, and sodium bicarbonate were also from C.S. Company, Haifa, Israel. The Cayman COX (human) Inhibitor Screening Assay Kit (Item # 701230) was included. RPMI 1640 culture medium and DPPH (catalog # 224448) were sourced from Sigma Aldrich, Germany. Silica gel (Merck, New Jersey, NJ, USA, 230–400 mesh) was used for flash chromatography with positive air pressure. Solvent evaporation was compelted using a Rota Vapor (Heidolph, Schwabach, Germany). NMR analysis was performed on a Bruker Avance 500 spectrometer at Jordan University, with chemical shifts in ppm and coupling constants in Hz. An Accumax Variable (Accumax Lab Devices Pvt Ltd., Gandhinagar, India) micropipette from the UK was used for pipetting, and the Unilab (Belgrade, Serbia) microplate reader 6000 read the Cayman ELISA kit. Sigma-Aldrich (Missouri, MO, USA) supplied porcine pancreatic lipase, starch, acarbose, PNPP (p-nitrophenyl palmitate), and orlistat, while MP Biochemicals (Illkirch, France) provided porcine pancreatic α-amylase.

### 3.2. Statistical Analysis and IC_50_ Calculations

The tests were carried out in triplicate. The results were reported as means ± standard deviation (SD). The IC_50_ value of the biological tests was calculated based on the inhibition percentage and the test material concentration. These values were plotted against the corresponding concentrations to create a dose-response curve. The IC_50_ value is obtained through nonlinear regression analysis or interpolating from the curve.

### 3.3. Chemical Synthesis and Characterization of the Products

The synthetic procedures, enzyme screening, and anticancer activity assessments were performed in the laboratories of An-Najah National University. The NMR (Bruker, Rheinstetten, Germany) measurements were conducted at the University of Jordan utilizing a Bruker Avance 400 instrument, as shown in the Supplementary Material. The compounds were initially synthesized in two stages: the tertiary alcohols were initially prepared, followed by their coupling with L-cysteine [47].

#### 3.3.1. Synthesis of Tertiary Alcohols

Compounds **1** and **2** were prepared by the standard Grignard reaction using phenyl or benzyl Grignard reagents added to the ketone form of trifluoromethyl thioxanthene. This procedure is illustrated in Scheme 1.

9-phenyl-2-(trifluoromethyl)-9H-thioxanthen-9-ol; compound **1**:

To obtain compound **1**; 2-(Trifluoromethyl) thio-xanthan-9-one (225 mg, 1 mmol) with phenyl magnesium chloride (268.35 µL, 2 mmol), DCM (5 mL). A pale-yellow powder was obtained (180 mg, 80% yield). R_f_: 0.45 (ethyl acetate/hexane (1:2)) M.P: 195–197 °C. IR: ATR, υ_max_ (cm^−1^): 3126.7 (OH stretch for alcohol). ^1^H NMR (500 MHz): δ (CDCl_3_): 2.73 (1H, s, -OH); 6.96 (2H, d, J = 8.0 Hz, Ar); 7.15–7.17 (3H, m, Ar), 7.24–7.30 (4H, m, Ar), 7.36–7.43 (3H, m, Ar). ^13^C NMR: (DMSO-d_6_) δ ppm: 116.62; 117.15; 120.61; 122.61; 122.93; 123.58; 124.14; 124.22; 125.58; 127.13; 127.86; 128.51; 129.10; 129.67; 137.41, 150.71; 152.22

9-benzyl-2-(trifluoromethyl)-9H-thioxanthen-9-ol; compound **2**:

To obtain compound **2**; 2-(Trifluoromethyl) thio-xanthan-9-one (225 mg, 1 mmol) with benzyl magnesium chloride (292.7 µL,2 mmol), DCM (5 mL). A pure yellow powder was obtained (180 mg, 48.4% yield). R_f_: 0.58 (ethyl acetate/hexane (1:2)), M.P:177–1179 °C. IR: ATR, υ_max_ (cm^−1^): 34.12 (OH stretch for alcohol). ^1^H NMR (500 MHz): δ (CDCl_3_): 2.46 (1H, s, –OH); 3.16 (2H, s, -CH_2_; 6.40 (2H, d, J = 8.0 Hz); 6.93 (4H, m, Ar); 7.15 (2H, t, Ar); 7.63 (2H, d, J = 7.5 Hz, Ar). ^13^C NMR: (DMSO-d_6_) δ ppm: 68.64; 115.78; 123.29; 125.5; 126.23; 127.84; 128.48; 128.51; 128.63; 128.76; 130.01; 149.07; 149.68.

#### 3.3.2. General Synthesis of Thioxanthene Analogues

In a reaction vessel, 0.5 mL of acetic acid served as the solvent, into which 1 mmol of L-cysteine and 1 mmol of the corresponding tertiary alcohol were dissolved. Boron trifluoride diethyl etherate, acting as a catalyst, was added dropwise to the reaction mixture while maintaining a temperature of 0 °C. The mixture was agitated for two hours at this temperature to facilitate the reaction. Progress was monitored using thin-layer chromatography (TLC). To terminate the reaction, 1.5 mL of a 10% sodium acetate solution and 1.5 mL of water were added. The resulting precipitate was filtered from the reaction mixture and rinsed with water. The filtered precipitate underwent an additional rinse with diethyl ether before being dried in a vacuum oven at 40 °C for twenty-four hours. The final product, L-cysteine derivatives, was obtained after drying and set aside for further characterization and analysis.

S-(9-benzyl-2-(trifluoromethyl)-9H-thioxanthen-9-yl)-L-cysteine; compound **3**:

To obtain compound **3**; L-Cysteine (121 mg,0.48 mmol) with 9-benzyl-2-(trifluoromethyl)-9H-thioxanthen-9-ol; compound **2** (358.4 mg, 1 mmol), boron trifluoride diethyl etherate (207 µL,1.68 mmol). A pure white powder was obtained (98 mg, 20.6% yield). R_f_: 0. 31 (DCM/ MeOH (9:1)), M.P: 243–245 °C IR: ATR, υ_max_ (cm^−1^): 3366.15 (OH stretch for carboxylic acid), 1701 (C = O stretch). ^1^H NMR (500 MHz): δ (DMSO-d_6_): 2.63 (1H, dd, J = 11.8/10.0 Hz); 2.71 (1H, dd, J = 11.8/3.9 Hz); 3.40 (2H, dd, J = 9.7/3.9 Hz); 7.28–7.30 (5H, m, Ar); 7.45–7.50 (5H, m, Ar); 7.65 (2H, d, J = 7.6 Hz). The protons of the NH_2_ and –OH were too broad to be observed. ^13^C NMR: (DMSO-d_6_) δ ppm: 31.74; 52.99; 53.34; 115.51; 115.61; 121.85; 122.30; 123.30; 123.37; 126.32; 127.23; 128.75; 128.80;129.46; 129.76; 135.74; 150.4; 151.05; 167.95.

Synthesis of S-(9-phenyl-9H-thioxanthen-9-yl)-L-cysteine; compound **4**:

To obtain compound **4**; L-Cysteine (121 mg,1 mmol) with 9-phenyl-2-(trifluoromethyl)-9H-thioxanthen-9-ol; compound **1** (372 mg, 1 mmol), boron trifluoride diethyl etherate (207 µL,1.68 mmol). A pure yellow powder was obtained (96 mg, 21.3% yield). R_f_: 0.15 (DCM/ MeOH (9:1)), M.P: 229–231 °C. IR: ATR, υ_max_ (cm^−1^): 3355.5 (OH stretch for carboxylic acid), 1650.06 (C = O stretch). ^1^H NMR (500 MHz): δ (DMSO-d_6_): 2.69 (1H, dd, J = 12.1/11.0 Hz; 2.85 (1H, dd, J = 12.1/4.2 Hz; 5.5 (1H); 7.13–7.17 (5H, m, Ar); 7.30–7.34 (3H, m, Ar); 7.47 (2H, d, J = 7.6 Hz, Ar); 7.61 (2H, d, J = 7.6 Hz, Ar). The protons of the NH_2_ and –OH were too broad to be observed. ^13^C NMR: (DMSO-d_6_) δ ppm: 32.29; 53.08; 55.15; 116.01; 116.10; 123.57; 123.61; 127.14; 128.37; 128.79; 128.87; 130.03; 145.32; 150.24; 150.34; 167.85.

### 3.4. Antioxidant Activity

To evaluate four synthesized compounds and Trolox (as a positive control), a 1 mg/mL concentration in methanol was first made from phenyl alcohol. The prepared solution was produced in concentrations of 1, 2, 3, 5, 7, 10, 20, 30, 50, and 80 g/mL. The DPPH reagent was then diluted in 0.002% *w*/*v* methanol and combined in a 1:1:1 (*v*/*v*/*v*) ratio with the previously produced working concentrations. A blank solution of 100% methanol was used. All solutions were incubated at room temperature for 30 min in a dark environment. Their absorbance values were then determined using a UV-visible spectrophotometer set at a wavelength of 517 nm. The antioxidant capacity of each chemical compound fraction and Trolox were calculated using the following formula:

% inhibition = ((Abs blank − Abs Sample)/Abs Blank) × 100

where:

Abs blank: Absorbance value of the control reaction containing all reagents except the synthesized compound.

Abs sample: The absorbance value of the synthesized compound [48].

### 3.5. α-Amylase Inhibition Assay

Each product was initially dissolved in a few milliliters of 10% DMSO and then further diluted in a buffer solution (0.02 M Na_2_HPO_4_/NaH_2_PO_4_, 0.006 M NaCl, at pH 6.9) to prepare stock solutions at a concentration of 1000 µg/mL. From these stock solutions, further dilutions were made to obtain concentrations of 20, 50, and 70 µg/mL, using 10% DMSO as a diluent. A porcine pancreatic α-amylase enzyme solution was prepared at 2 units/mL concentration in 10% DMSO. For the working solutions, 0.2 mL of the enzyme solution was mixed with 0.2 mL of each product, and the mixture was incubated for 10 min at 30 °C. Following the incubation, 0.2 mL of a freshly prepared 1% starch aqueous solution was added to each working solution, which was then incubated for an additional 3 min. The reaction was halted by adding 0.2 mL of dinitrosalicylic acid (DNSA) yellow color reagent. Each working solution was diluted with 5 mL of distilled water and boiled for 10 min in a water bath at 90 °C. After cooling the mixture to room temperature, the absorbance was measured at 540 nm. The blank control was prepared similarly but replaced the product fraction with 0.2 mL of the buffer as mentioned above. Acarbose was used as the standard reference and was subjected to the same experimental procedures. The α-amylase inhibitory activity was calculated using the following equation [49]:

% of α amylase inhibition = (AB − AE)/AB × 100%

### 3.6. Inhibition Assay of Pancreatic Lipase Enzyme

A stock solution of 1 mg/mL (1000 μg/mL) was prepared for each product using 10% DMSO. This stock solution created five different concentrations at 20, 50, and 70 μg/mL. A fresh stock solution of pancreatic lipase enzyme was prepared immediately before use. For the preparation of the substrate, a stock solution of p-nitrophenyl butyrate (PNPB) was made by dissolving 20.9 mg of PNPB in 2 mL of acetonitrile. In the experiment, 0.1 mL of the 1 mg/mL porcine pancreatic lipase solution was added to test tubes containing 0.2 mL of each of the various concentrations of the products. The mixtures were then brought to a final volume of 1 mL by adding a Tris-HCl solution (pH 7.4) and were incubated at 25 °C for 15 min. After the incubation period, 0.1 mL of the PNPB solution was added to each test tube, and the mixtures were incubated again for 30 min at 37 °C. The activity of pancreatic lipase was measured by assessing the hydrolysis of p-nitrophenyl butyrate to p-nitrophenol at a wavelength of 410 nm using a UV-visible spectrophotometer. The same procedure was followed for orlistat, which served as the standard reference. The lipase enzyme inhibitory potential was measured using the following equation:

I (%) = ([Aa − Ats]/AB) × 100%

where, I (%), is the percentage inhibition of lipase enzyme, AB is the recorded absorbance of the blank, and Ats is the absorbance of the tested sample [50].

### 3.7. Cytotoxicity Procedure

RPMI-1640 medium was used to culture cervical adenocarcinoma (HeLa) cancer cells, supplemented with 10% fetal bovine serum, 1% penicillin/streptomycin, and 1% L-glutamine. The HeLa cells were grown in a humidified environment at 37 °C with a 5% CO_2_ atmosphere. A 96-well plate was employed for seeding the cells at a density of 2.6 × 10^4^ cells per well. Following a 24-h incubation, the cells were treated with various concentrations of the tested (AO) fractions. Cell viability was assessed using the Cell Titer 96^®^ Aqueous One Solution Cell Proliferation (MTS) assay, following the manufacturer’s instructions (Promega Corporation, Wisconsin, WI, USA). Briefly, at the end of the treatment, 20 µL of MTS solution was added to each well containing 100 µL of media, and the cells were incubated at 37 °C for 2 h. The absorbance was measured using a UV-visible spectrophotometer at a wavelength of 490 nm [51].

### 3.8. COX Inhibition Activity

Our synthesized compounds’ COX-1 and COX-2 inhibitory activities were evaluated using the COX (human) Inhibitor Screening Assay Kit supplied by Cayman Chemicals (catalog no. 701230, Ann Arbor, MI, USA). The preparation of reagents and testing procedures were conducted according to the manufacturer’s recommendations. Two concentrations of the inhibitors (10 µM and 50 µM) were dissolved in a minimal amount of dimethyl sulfoxide (DMSO) and incubated with a mixture of COX-1 or COX-2 enzyme and heme in a diluted reaction buffer for 10 min at 37 °C. The reaction was initiated by adding 10 µL of arachidonic acid, followed by a two-minute incubation at 37 °C. The reaction was halted by adding 30 µL of stannous chloride solution to each reaction tube and incubating for 5 min at room temperature. The resulting PGF2α, produced by the COX reactions, was quantified using an enzyme-linked immunosorbent assay (ELISA). The 96-well plate was covered with plastic film and incubated for 18 h at room temperature on an orbital shaker. After incubation, the plate was rinsed five times with a wash buffer. Then, Ellman’s reagent (200 µL) was added, and the plate was incubated for approximately 60–90 min at room temperature until the absorbance of the blank well was in the range of 0.3–0.8 at 405 nm. The plate was read using a Unilab Microplate Reader 6000. The inhibitory percentage was calculated for each concentration tested against the control. The IC_50_ values were derived from the concentration-inhibition response curve, and the selectivity index (SI) was calculated by dividing the IC_50_ for COX-2 by the IC_50_ for COX-1 [52].

## 4. Conclusions

This study successfully synthesized and characterized novel trifluoromethyl thioxanthene derivatives by incorporating L-cysteine moieties to enhance their biological activity. The chemical structures of the synthesized compounds were confirmed using various spectroscopic techniques, including infrared (IR) and nuclear magnetic resonance (NMR) analysis. Biological evaluations demonstrated that the synthesized compounds exhibited promising antioxidant properties. The most active compound was compound **3**, which had a DPPH radical scavenging activity of 46.6% at 80 μg/mL. Notably, compound **4** has a similar antioxidant activity, suggesting that the benzyl substitution in its structure enhanced electron-donating properties, further strengthening antioxidant potential. In addition, the α-amylase and pancreatic lipase inhibition assays revealed significant enzyme inhibition, with the most potent derivative showing an α-amylase IC_50_ inhibition of 60.2 ± 0.8 µM and lipase IC_50_ inhibition of 100.6 ± 7.3 µM, suggesting its potential role in the management of metabolic disorders such as diabetes and obesity. Furthermore, cytotoxicity assessments against HeLa cancer cells indicated notable anticancer activity, with IC_50_ values ranging from 87.8 to 197 nM, reflecting a dose-dependent response. The COX inhibition assays revealed selective COX-2 inhibition, with the most effective compound **4** displaying an IC_50_ inhibition of 6.5 ± 0.77 µM, with COX2 selectivity of 1.6. This suggests potential anti-inflammatory properties of compound **4** with reduced gastrointestinal side effects. Overall, this study underscores the pharmaceutical potential of the newly synthesized trifluoromethyl thioxanthene derivatives. Further investigations, including SAR, in vivo studies, and mechanistic analyses, are warranted to further explore their therapeutic potential.

## Figures and Tables

**Table 1:** The results of % inhibition of antioxidant activity.

Conc. (µg/mL)	Trolox	Comp 1	Comp 2	Comp 3	Comp 4
1	30.5 ± 2.2	7.1 ± 1.2	36.3 ± 2.4	31.3 ± 2.3	30.5 ± 1.2
2	23.6 ± 1.3	7.6 ± 2.3	37.3 ± 3.9	36.4 ± 3.5	36.4 ± 2.5
3	61.4 ± 5.3	8.2 ± 1.5	39.3 ± 4.2	39.9 ± 3.6	39.4 ± 1.5
5	90.7± 11.3	8.6 ± 3.1	39.5 ± 3.4	39.9 ± 4.1	41.2 ± 3.1
7	95.3 ± 10.7	8.9 ± 2.3	39.7 ± 3.6	40.1 ± 3.8	41.6 ± 3.6
10	95.9± 9.5	9.3 ± 1.6	40.1 ± 4.9	40.2 ± 5.1	41.9 ± 2.8
20	96.7 ± 12.5	9.9 ± 2.5	40.7 ± 2.5	40.4 ± 5.2	42.2 ± 2.4
30	97.2 ± 8.5	12.6 ± 3.2	40.7 ± 3.8	41.9 ± 4.9	43.3 ± 6.1
50	98.1± 9.2	12.9 ± 2.6	41.6 ± 5.1	42.9 ± 3.2	45.5 ± 5.1
80	98.9± 11.8	16.3 ± 4.3	42.2 ± 3.9	46.6 ± 2.4	45.6 ± 2.6

**Table 2:** COX inhibition activity for the synthesized compound.

Compounds	COX-1 IC_50_ (nM)	COX-2 IC_50_ (nM)	COX-2 Selectivity
Comp (**1**)	40.2 ± 0.41	27.1 ± 0.6	1.5
Comp (**2**)	14.0 ± 0.64	74.1 ± 0.48	0.2
Comp (**3**)	32.5 ± 0.53	25.9 ± 0.45	1.3
Comp (**4**)	10.1± 1.30	6.5 ±0.77	1.6

## Data Availability

All data generated or analyzed during this study are included in this published article.

## Supplementary Materials

The following supporting information can be downloaded at: https://www.mdpi.com/article/10.3390/ph18040561/s1, Supplementary Material: the NMR analysis of each compound.
