# Supplementary material for: Bioactivity of Synthesized Trifluoromethyl Thioxanthone Analogues
Source: Pharmaceuticals (Basel). 2025 Apr 11;18(4):561. doi: 10.3390/ph18040561 (PMC12030165; doi:10.3390/ph18040561)

Supplementary Material

Bioactivity of Synthesized Trifluoromethyl Thioxanthone Analogues

Murad Abualhasan *, Hussein Haider, Ahmad Odeh and Amer Daraghmeh

Faculty of Pharmacy, An-Najah National University, Nablus 00970, Palestine; hussienhaidr0597184299@gmail.com (H.H.); ph.ahmad.o.r@gmail.com (A.O.); amersalehdd@gmail.com (A.D.)
* Correspondence: Correspondence: m_abualhasan@najah.edu

Figure Compound 1: 9-phenyl-2-(trifluoromethyl)-9H-thioxanthen-9-ol 2

Figure Compound 2: 9-benzyl-2-(trifluoromethyl)-9H-thioxanthen-9-ol 4

Figure Compound 3: S-(9-benzyl-2-(trifluoromethyl)-9H-thioxanthen-9-yl)-L-cysteine 6

Figure Compound 4: Synthesis of S-(9-phenyl-9H-thioxanthen-9-yl)-L-cysteine 7

Figure Compound 1: 9-phenyl-2-(trifluoromethyl)-9H-thioxanthen-9-ol

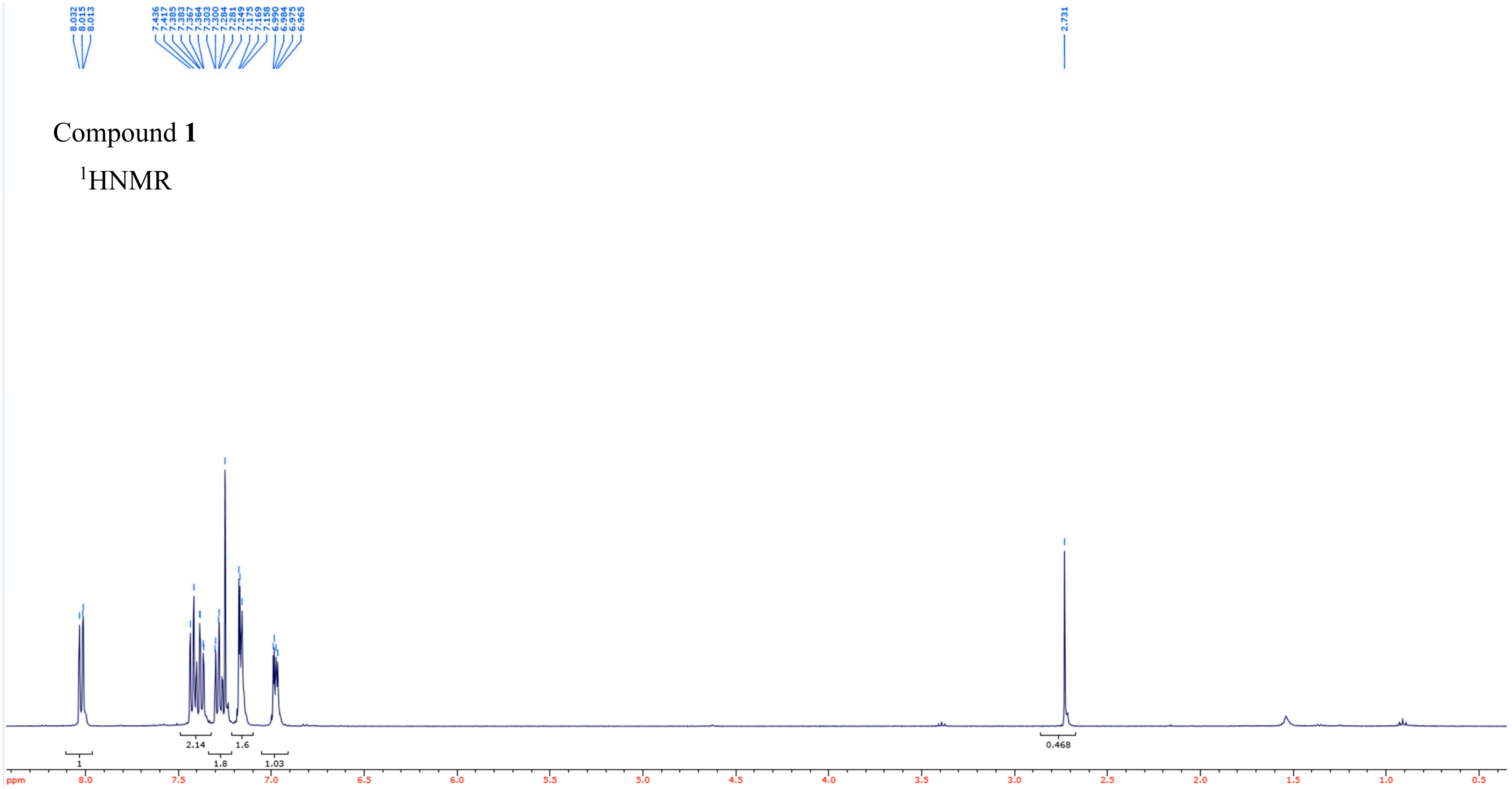

^{13}C NMR

Compound **1**

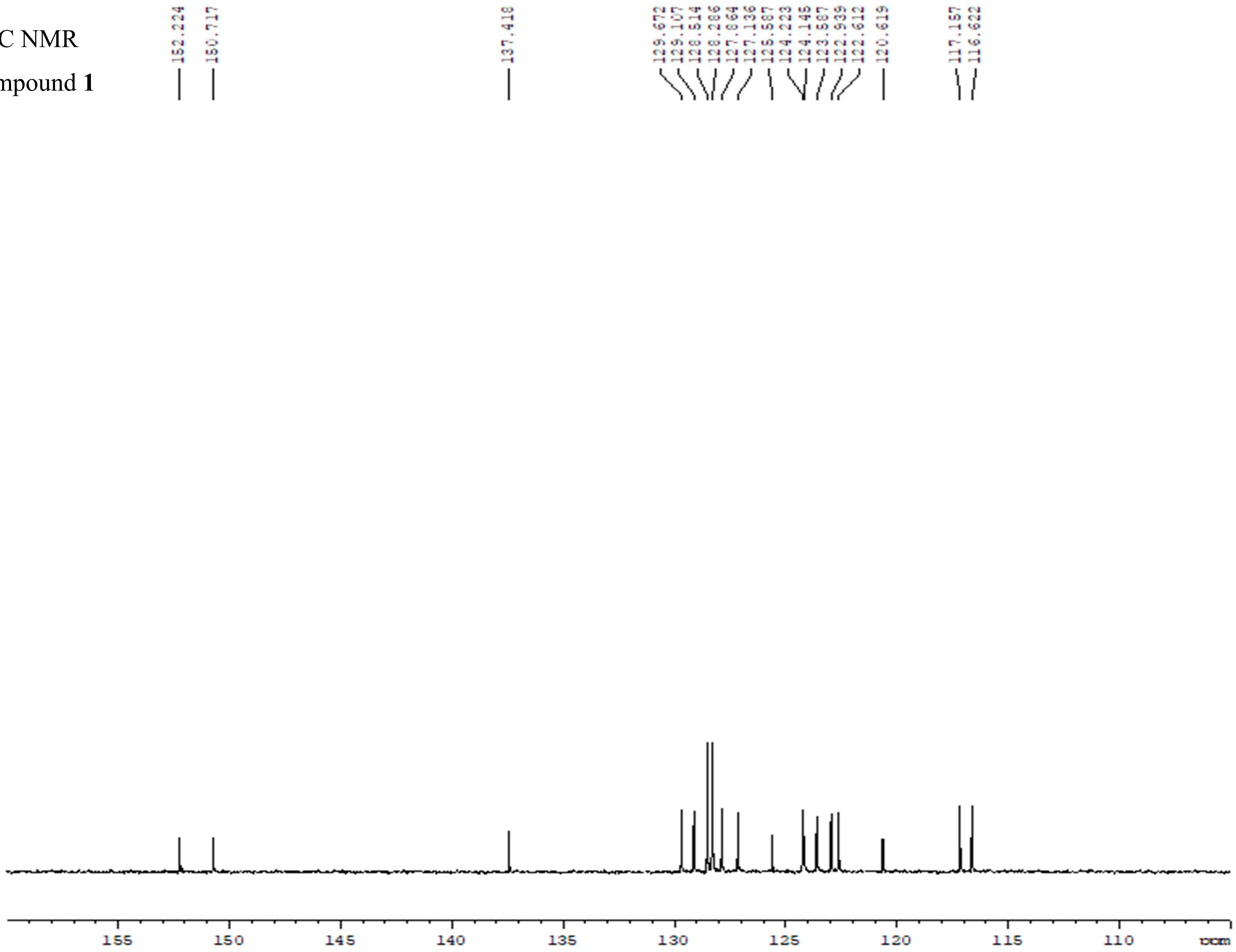

Figure Compound 2: 9-benzyl-2-(trifluoromethyl)-9H-thioxanthen-9-ol

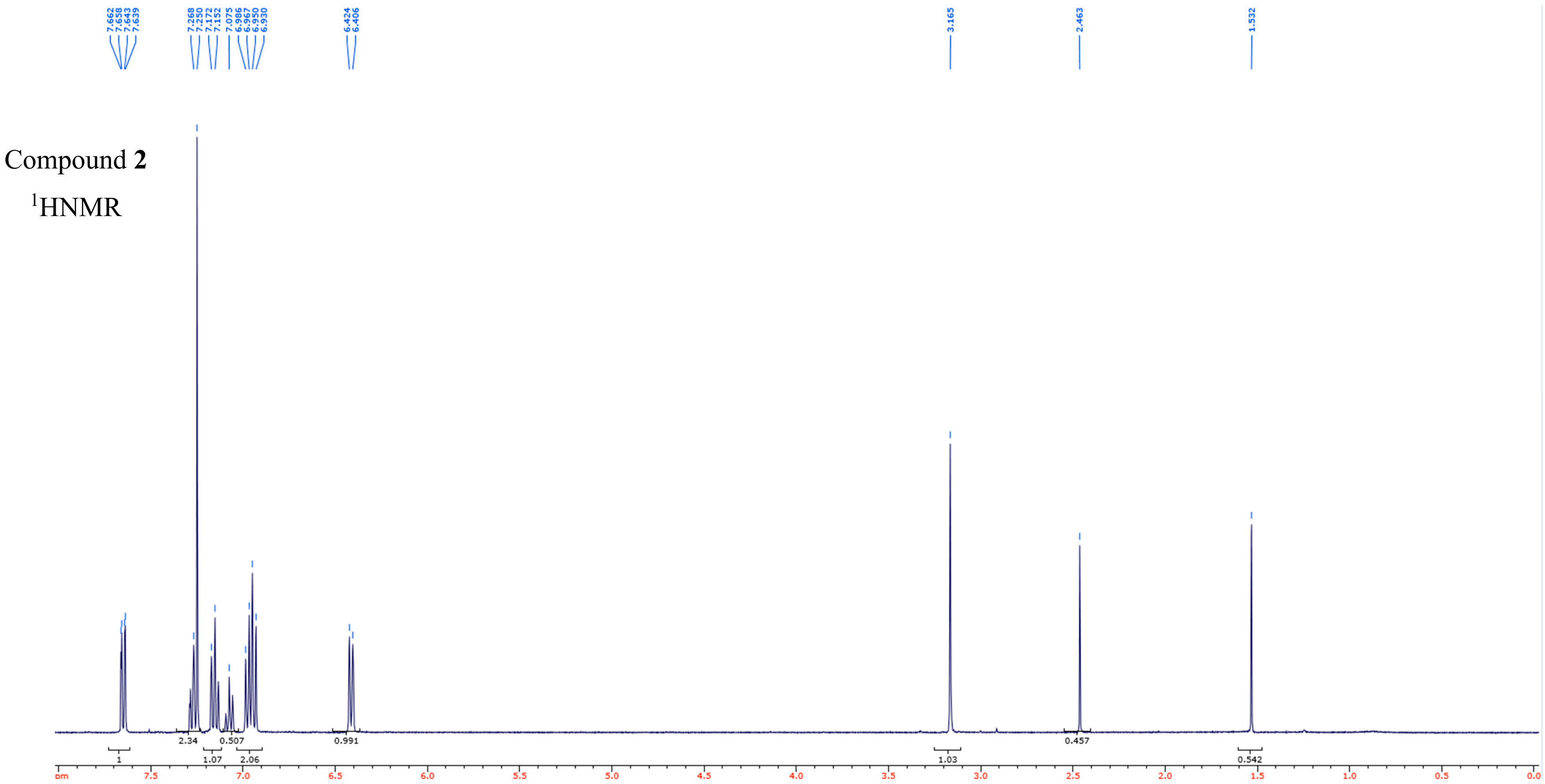

^{13}C NMR

Compound **2**

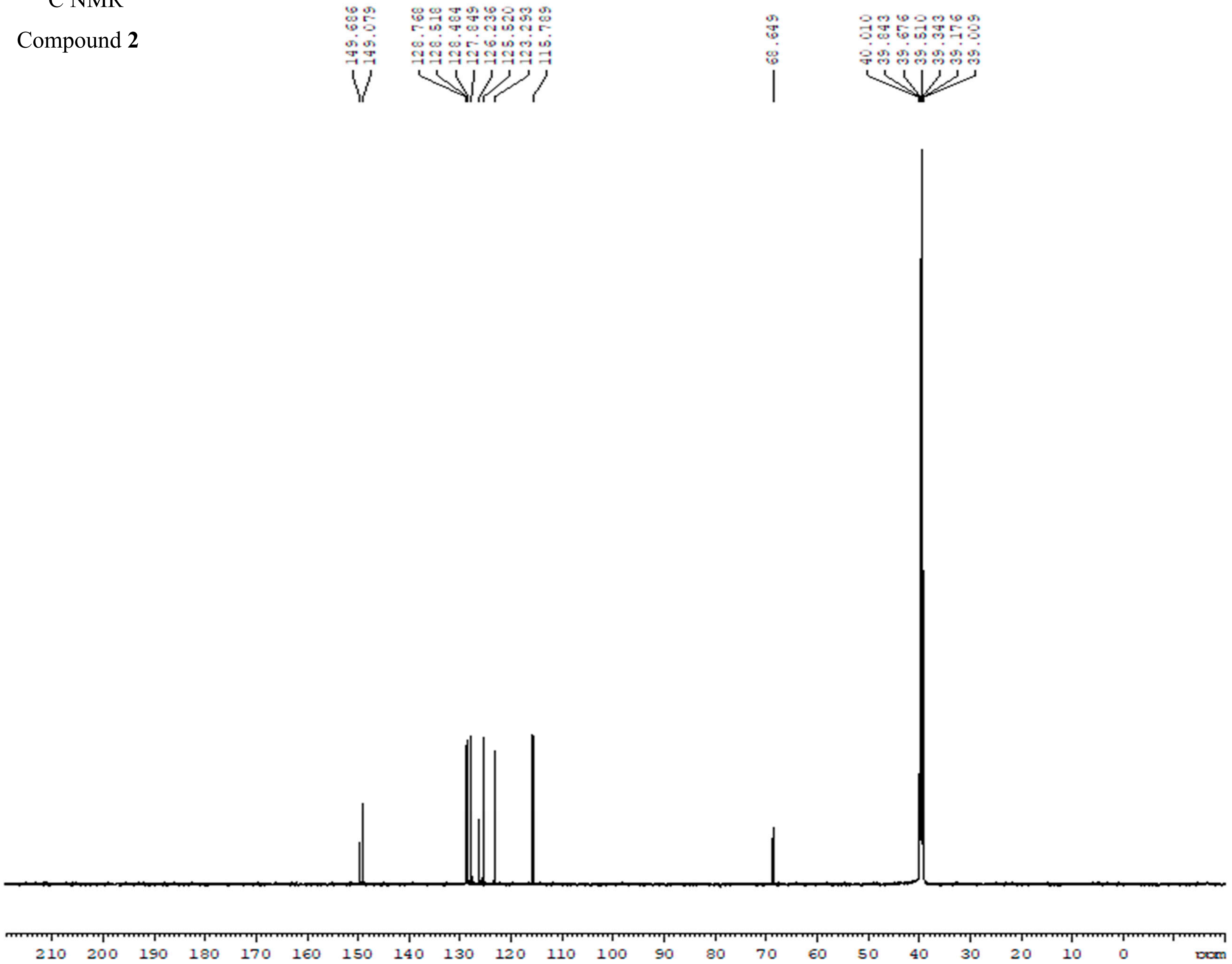

Figure Compound 3: S-(9-benzyl-2-(trifluoromethyl)-9H-thioxanthen-9-yl)-L-cysteine

Compound **3**

^{1}HNMR

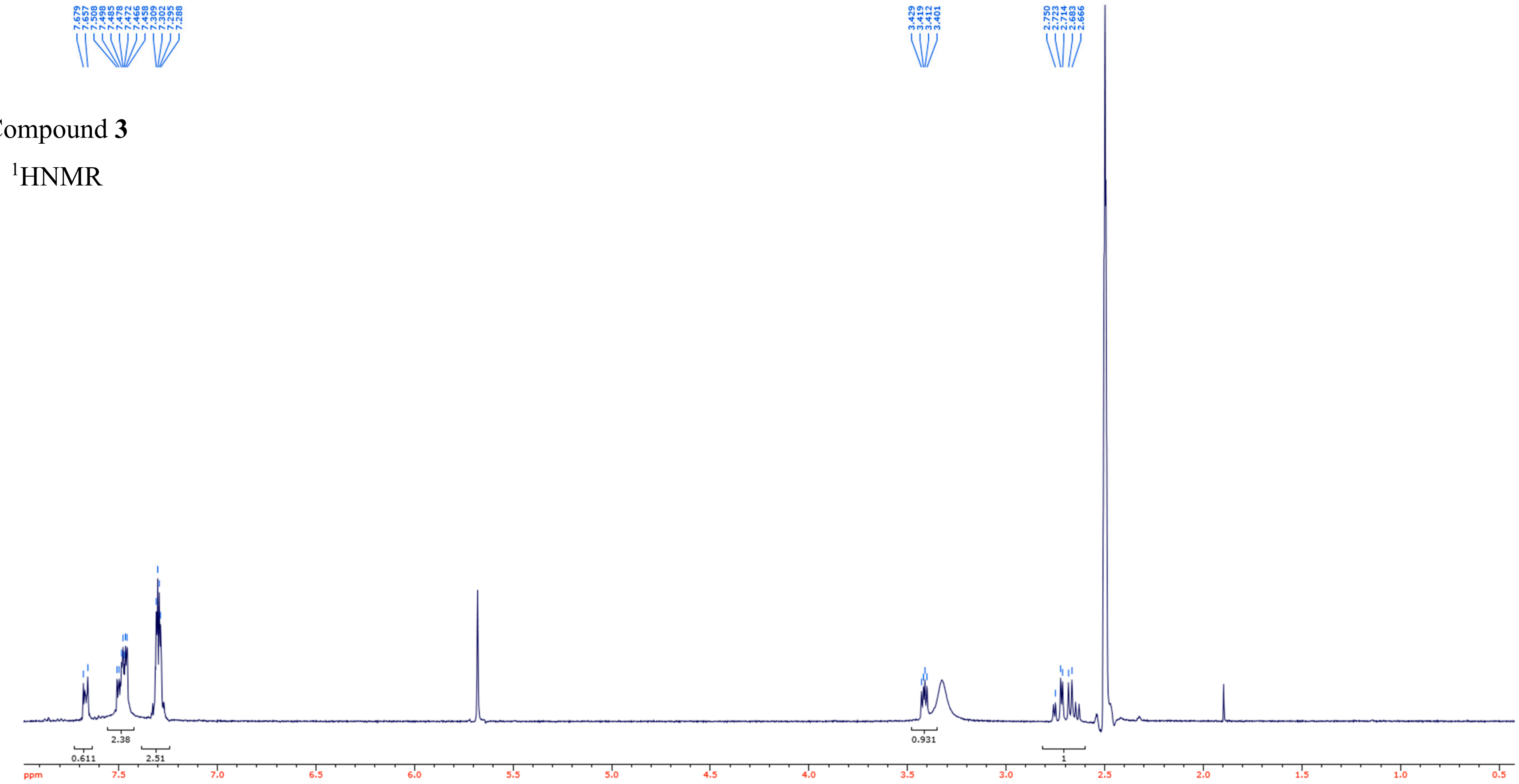

^{13}C NMR

Compound **3**

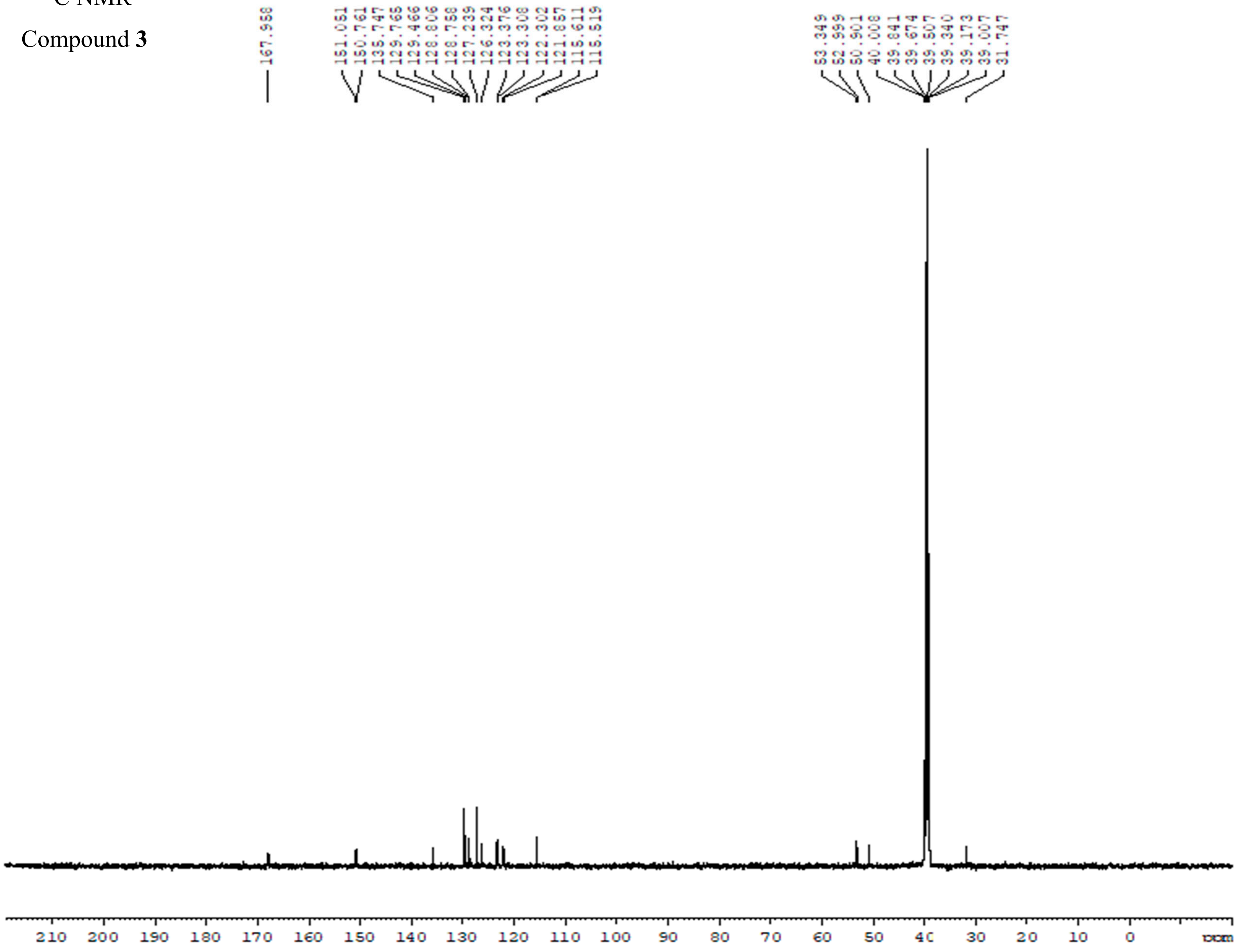

Figure Compound 4: Synthesis of S-(9-phenyl-9H-thioxanthen-9-yl)-L-cysteine

Compound **4**

^{1}HNMR

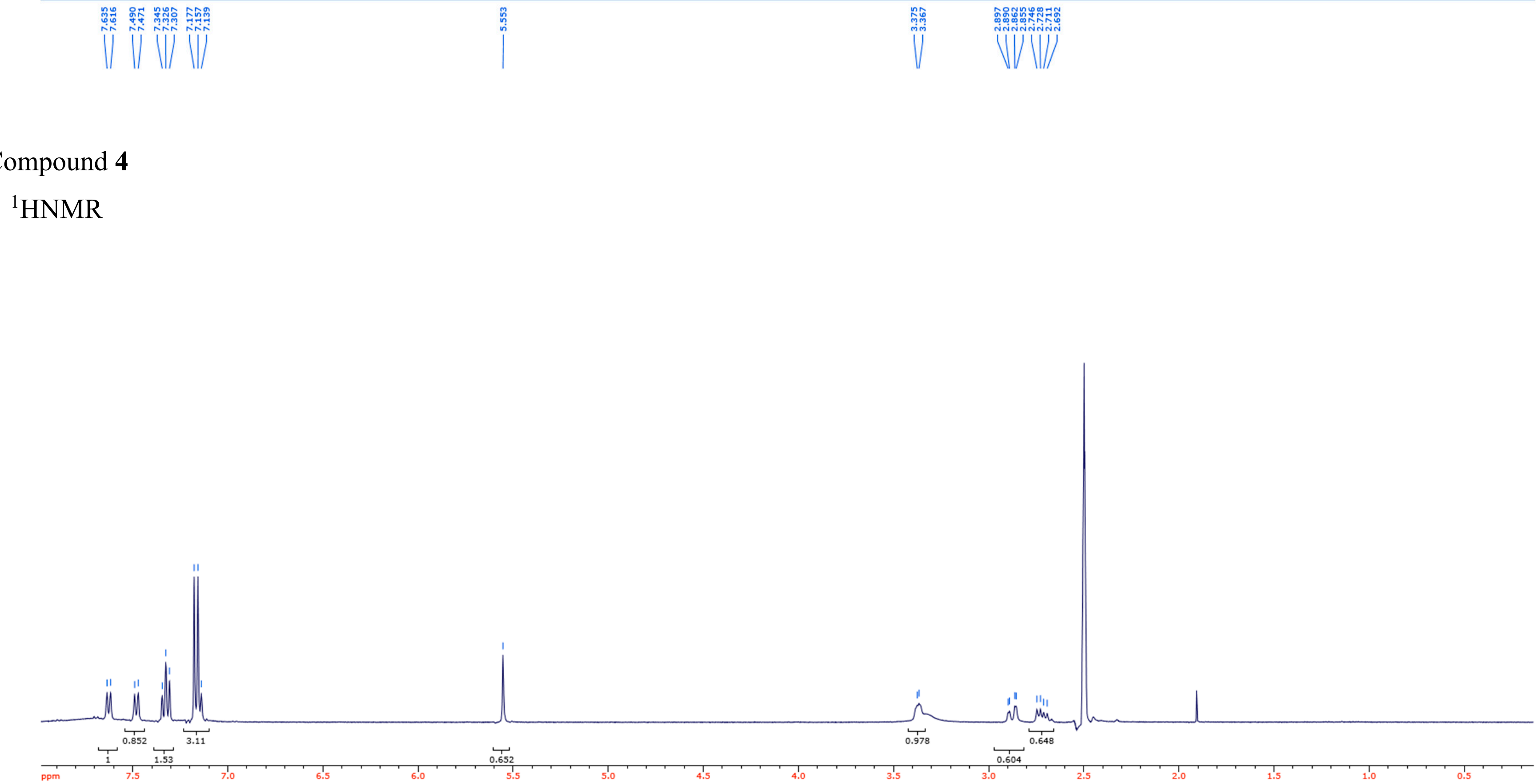

^{13}C NMR

Compound **4**

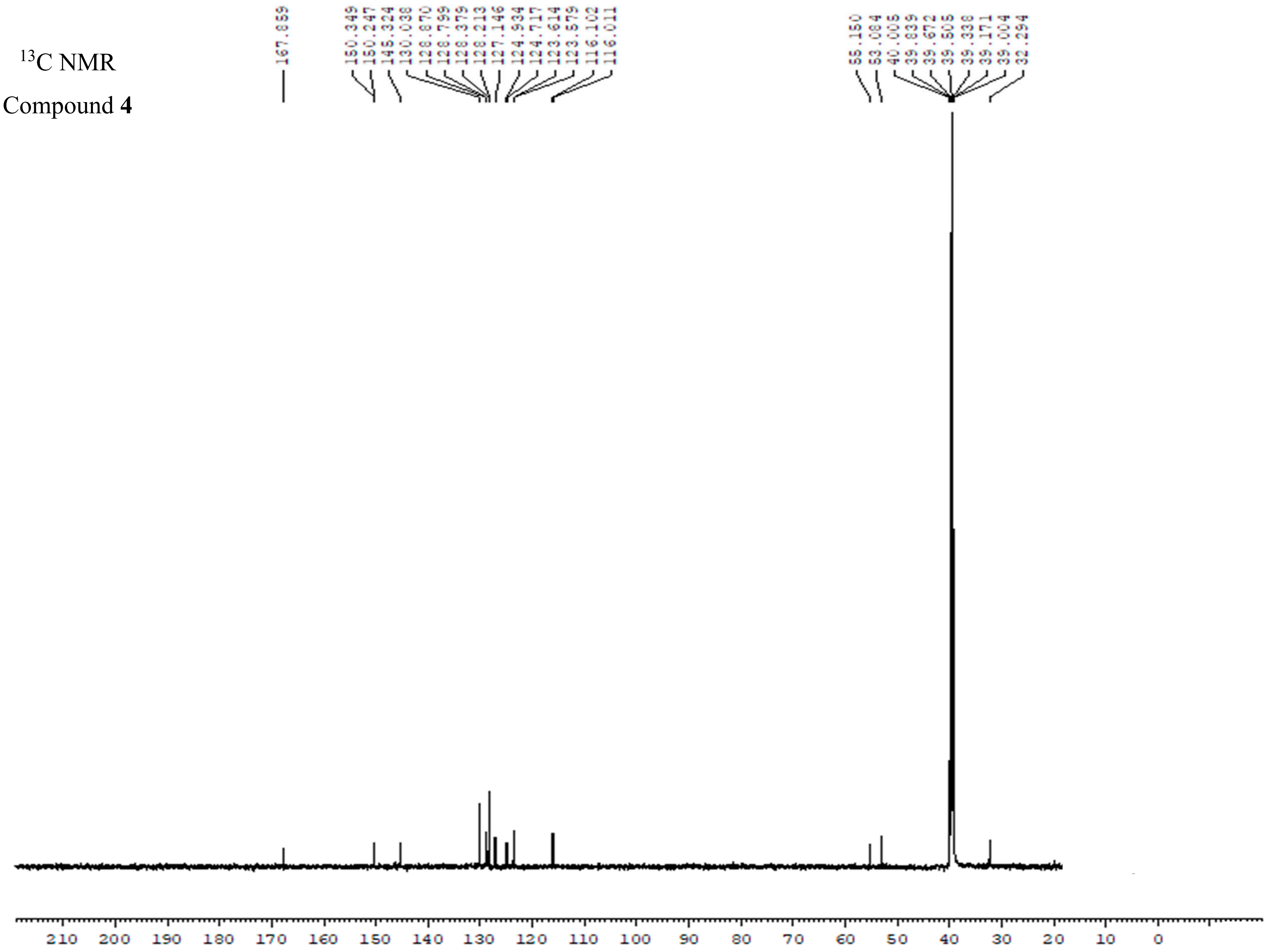

Supplement: Supplementary file 1 [file pharmaceuticals-18-00561-s001.zip › pharmaceuticals-3518104-supplementary.pdf]
